# Anatomical Comparison of Antennal Lobes in Two Sibling *Ectropis* Moths: Emphasis on the Macroglomerular Complex

**DOI:** 10.3389/fphys.2021.685012

**Published:** 2021-08-12

**Authors:** Jing Liu, Kang He, Zong-xiu Luo, Xiao-ming Cai, Lei Bian, Zhao-qun Li, Zong-mao Chen

**Affiliations:** ^1^Key Laboratory of Tea Biology and Resource Utilization, Ministry of Agriculture, Tea Research Institute, Chinese Academy of Agricultural Science, Hangzhou, China; ^2^Institute of Insect Sciences, College of Agriculture and Biotechnology, Zhejiang University, Hangzhou, China

**Keywords:** glomeruli, macroglomerular complex, antennae backfill, antibody staining, *Ectropis obliqua*, *Ectropis grisescens*

## Abstract

*Ectropis obliqua* and *Ectropis grisescens* are two sibling moth species of tea plantations in China. The male antennae of both species can detect shared and specific sex pheromone components. Thus, the primary olfactory center, i.e., the antennal lobe (AL), plays a vital role in distinguishing the sex pheromones. To provide evidence for the possible mechanism allowing this distinction, in this study, we compared the macroglomerular complex (MGC) of the AL between the males of the two species by immunostaining using presynaptic antibody and propidium iodide (PI) with antennal backfills, and confocal imaging and digital 3D-reconstruction. The results showed that MGC of both *E. obliqua* and *E. grisescens* contained five glomeruli at invariant positions between the species. However, the volumes of the anterior-lateral glomerulus (ALG) and posterior-ventral (PV) glomerulus differed between the species, possibly related to differences in sensing sex pheromone compounds and their ratios between *E. obliqua* and *E. grisescens*. Our results provide an important basis for the mechanism of mating isolation between these sibling moth species.

## Introduction

The sophisticated olfactory system of insect species is a key physiological feature involved in behavioral decisions. In insects, olfactory cues are detected by olfactory receptor neurons (ORNs) housed in the antennal sensilla and projected to the antennal lobe (AL). The AL is the primary olfactory center of the brain responsible for integrating complex olfactory information (Homberg et al., 1989; Kuebler et al., 2012). Glomerulus, as the olfactory functional unit, is a subcompartment of the AL and is formed by dense synaptic and recipient dendrites from the ORNs that convey olfactory information from the antenna (Skiri et al., 2005; Yan et al., 2019). The number of glomeruli is species-specific, and their total volume and position are closely related to the complexity of odorant recognition (Berg et al., 2014; Wu et al., 2015).

The macroglomerular complex (MGC) specifically translates sex pheromone information and is a male-specific structure in moth species. Although some species possess a single MGC component (Varela et al., 2009), most moth species have three to four MGC glomeruli (Hansson et al., 1994; Lee et al., 2006; Namiki et al., 2014; Nirazawa et al., 2017; Jiang et al., 2019; Yan et al., 2019; Dong et al., 2020). Different groups of moth species display different anatomical features. Noctuid species normally possess one large MGC at the entrance of the antennal nerves and smaller satellite glomeruli (Berg et al., 2014). However, in *Bombycinae* species, the cumulus coexists with the toroid in different volume combinations (Namiki et al., 2014).

Sibling moth species generally have the same number of MGC glomeruli. Nevertheless, the position of the MGC glomeruli is slightly different in *Helicoverpa* species (Xu et al., 2016a,b), while the same number and position of MGC glomeruli are present in *Heliothine* species (Vickers and Christensen, 2003). The study of two *Helicoverpa* sibling species, *Helicoverpa armigera* and *H. assulta*, showed that different combinations of certain glomeruli positions and volumes in the MGC could reflect the ratio of the major and minor sex pheromone compartments, which play a vital role in sex pheromone discrimination (Wu et al., 2013).

According to their chemical structures, the sex pheromones emitted from female moths are classified into two main groups: Type I pheromones (75%) are composed of unsaturated compounds with a C10–C18 straight chain and a terminal functional group, such as hydroxyl, acetoxyl, or formyl groups; and Type II pheromones (15%) are composed of unsaturated hydrocarbons and epoxy derivatives with a C17–C23 straight chain. Type I pheromones have been identified from species within various families, such as Crambidae, Tortricidae, and Noctuidae; Type II pheromones are mainly produced by species within highly evolved insect groups, such as Geometridae, Lymantriidae, and Arctiidae moths (Touhara, 2013). However, most studies comparing the AL MGC of sibling moth species have focused on species producing Type I pheromones (Varela et al., 2011). With the rare exception of studies on single species, no anatomical or physiological comparison has been performed for the MGC of species producing Type II pheromones (Namiki et al., 2012).

*Ectropis obliqua* and *E. grisescens* (Lepidoptera, Geometridae) are two sibling herbivorous pest species of tea plantations. These two species co-occur in the tea plantations of southeast China and share similar sex pheromone components (Luo et al., 2017; Li et al., 2019). The sex pheromone components of *E. grisescens* females were identified as (Z,Z,Z)-3,6,9-octadecatriene (Z3,Z6,Z9-18:H) and (Z,Z)-3,9-cis-6,7-epoxy-octadecadiene (Z3,epo6,Z9-18:H) and those of *E. obliqua* were Z3,Z6,Z9-18:H, Z3,epo6,Z9-18:H, and (Z,Z)-3,9-cis-6,7-epoxy-nonadecadiene (Z3,epo6,Z9-19:H). These sex pheromones of both species are Type II and differences in sex pheromone compounds and their ratios play important roles in the mating isolation of these two sibling species (Touhara, 2013; Luo et al., 2017). Single sensilla recording (SSR) showed the tricoid sensilla type of both species can recognize all three components of the sex pheromones (Liu et al., 2019). However, the population density of the ORNs tuned to the minor component, i.e., Z3,Z6,Z9-18:H, differs between the two species. The reasons for this difference in interspecific sex pheromone recognition and how olfactory information is processed at the primary olfactory center level are still unknown. Therefore, in this study, we compared the anatomy of MGC in *E. obliqua* and *E. grisescens* to determine whether differences in glomeruli number, position, and volume between these two moth species can explain the mechanism of sex pheromone discrimination between them.

## Materials and Methods

Larvae of male *E. obliqua* were collected from a tea plantation belonging to the Tea Research Institute, Chinese Academy of Agricultural Science, Hangzhou City, Zhejiang Province, China (30.10°N, 120.5°E). Male and female *E. grisescens* larvae were collected from a Yuchacun Tea Co., Ltd. tea plantation in Shaoxing City, Zhejiang Province, China (29.56°N, 120.41°E). After species identification (Li et al., 2019), the larvae were separately reared on fresh tea shoots in a climate-controlled chamber at 25°C, with 60% relative humidity, and a photoperiod of 12:12 h (light:dark). On the second day after eclosion, adults of both species were selected for the experiment.

The presynaptic antibody SYNORF1 (Developmental Studies Hybridoma Bank, University of Iowa, Iowa City, IA, USA) was used for immunostaining to visualize the AL glomerular structure. Eight or nine male moths of both species were anesthetized on ice. Heads were separated from the body and brains were dissected in Ringer's solution. After fixation in 4% paraformaldehyde solution in phosphate-buffered saline (PBS; 0.01M, pH 7.4) overnight at 4°C, the brains were rinsed in PBS four times (20 min per rinse) and pre-incubated in 5% normal goat serum (Sigma Aldrich, St. Louis, MO, USA) in PBS containing 0.5% Triton X-100 (PBST; 0.01 M, pH 7.4) for 3 h at room temperature (20–25°C) to diminish the staining disturbance. Following incubation in the primary antibody SYNORF1 at 1:50 in PBST at 4°C for 7 days, the brains were rinsed in PBS six times (20 min per rinse) and then incubated in the secondary antibody, Cy™2-conjugated anti-mouse IgG (H + L) (Cy™2; Jackson Immuno Research, GE Healthcare Bio-Sciences Ltd., West Grove, PA, USA; dilution 1:150 in PBST) at 4°C for 5 days. After this period, the brains were rinsed in PBS six times (20 min per rinse), dehydrated in an ascending ethanol series, and mounted in methyl salicylate.

To mark the olfactory receptor neurons (ORNs) axons, backfills of the antennal nerve were performed using the neuronal tracer neurobiotin (Vector Laboratories, Burlingame, CA, USA). Neurobiotin backfilled antennal nerves were counterstained with propidium iodide (PI; MedChem Express, Monmouth Junction, NJ, USA). The neurobiotin concentration applied (1%) was the same used in similar studies on other insect species (Yan et al., 2019). The insect was fixed in a cone-shaped plastic tube exposing the head and the antennae out of one tip of the tube. Both antennae were cut at the base of the flagellum and the antennal nerve was exposed. The tip of a 2-mm long pipette was filled with the neurobiotin solution; one end of the tip was then sealed with Vaseline. The other end of the pipette tip, which covered the cut end of the antenna, was closed and immobilized with Vaseline. The cut antenna was placed into the pipette ensuring the cut end of the AL was immersed in the neurobiotin tracer solution. Whole preparations were incubated in a dark box with moist paper to avoid desiccation for 24–36 h at 4°C. Then, brains were dissected in Ringer's solution and fixated in 4% paraformaldehyde in PBS (0.1 M, pH 7.4) at 4°C for 1–2 days. For neurobiotin staining, brains were incubated in 2 μg/mL Alexa-488 Streptavidin solution in PBS (pH7.4; Yeason, Shanghai, China) for 1–2 days. Counterstaining was developed using 10 μg/mL PI solution for 30 min at room temperature (20–25°C).

Confocal laser scanning microscopy (LSM 510, META Zeiss, Jena, Germany) was used for AL tomography scanning (objectives: Plan-Neofluar 20 ×/0.5 L) at a step size of 0.3 μm. A 488-nm line of an argon laser was used to excite the Cy™2 antibody or Alexa-488 Streptavidin. The serial optical images were obtained after scanning at a resolution of 512 × 512 pixels. The 3D structure of the AL glomeruli was obtained from reconstructed confocal image stacks by using the AMIRA 5.3 software (Visage Imaging, Fürth, Germany). The volumes of individual glomeruli were measured by the “Tissue Statistics” tool and the quantitative data were imported to Microsoft Excel (Microsoft Corp., Redmond, WA, USA) for further processing.

Male *E. obliqua* and *E. grisescens* were used for presynaptic antibody staining and backfills combined with PI. Only backfills were applied to female *E. grisescens*.

The glomerular volume data were analyzed by one-away ANOVA and *t*-tests in SigmaPlot.

## Results

### Histology of the AL of *E. obliqua* and *E. grisescens*

**Figure 1:**
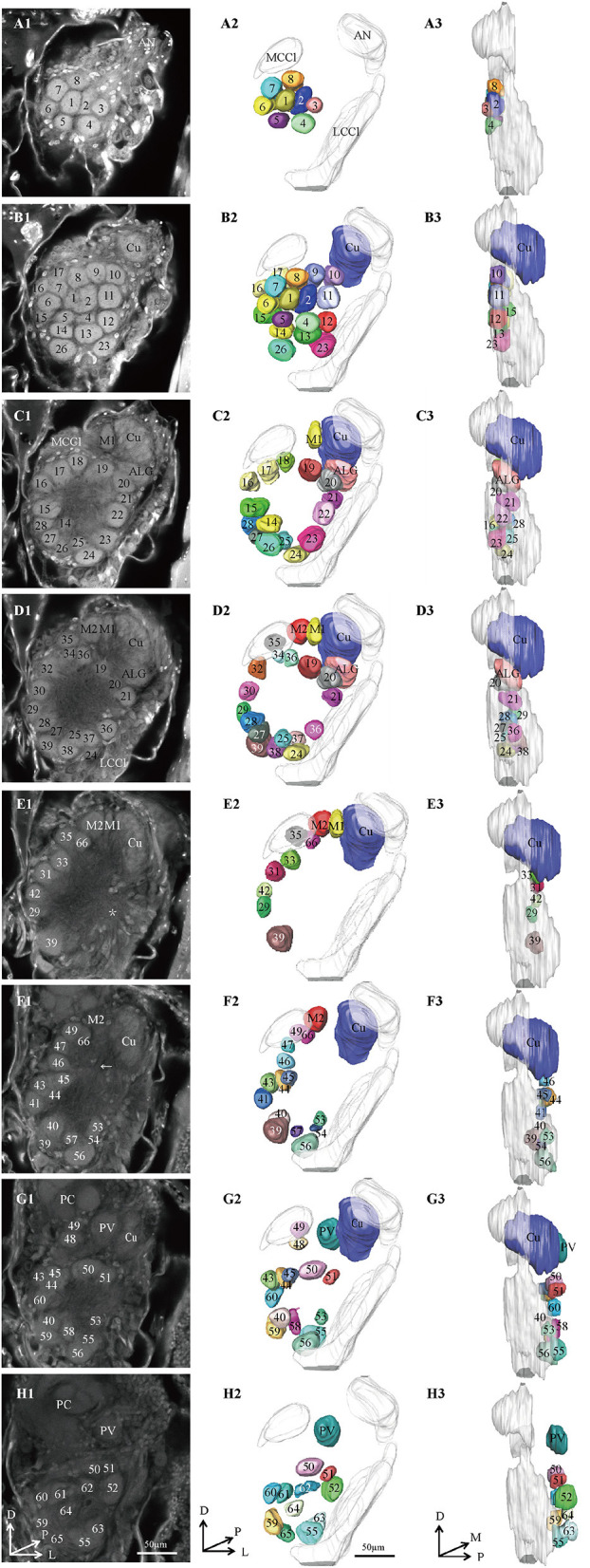
Confocal images and 3D reconstructions of the AL glomeruli of male E. obliqua in a frontal view. **(A1–H1)** confocal images of AL sections at different depths. The most anterior glomeruli, at 13 μm (A), 22 μm (B), 35 μm (C), 46 μm (D), 56 μm (E), 67 μm (F), 76 μm (G), and 92 μm (H). **(A2–H2)** 3D reconstructions of glomeruli from the confocal images shown in **(A1–H1)** (frontal view). **(A3–H3)** lateral view of the 3D reconstructions shown in **(A2–H2)** AN, antennal nerve; LCCl, lateral cell cluster; MCCl, medial cell cluster; PC, protocerebrum; Cu, cumulus; ALG, anterior-lateral glomerulus; M1, medial glomerulus 1; M2, medial glomerulus 2; PV, posterior-ventral glomerulus. The arrow indicates bundles of MCCl and the star indicates bundles of LCCl. A, anterior; D, dorsal; M, medial. Scale bars 50 μm. Images were drawn by AMIRA 5.3 and further enhanced by adobe illustrator CS 5.

**Figure 2:**
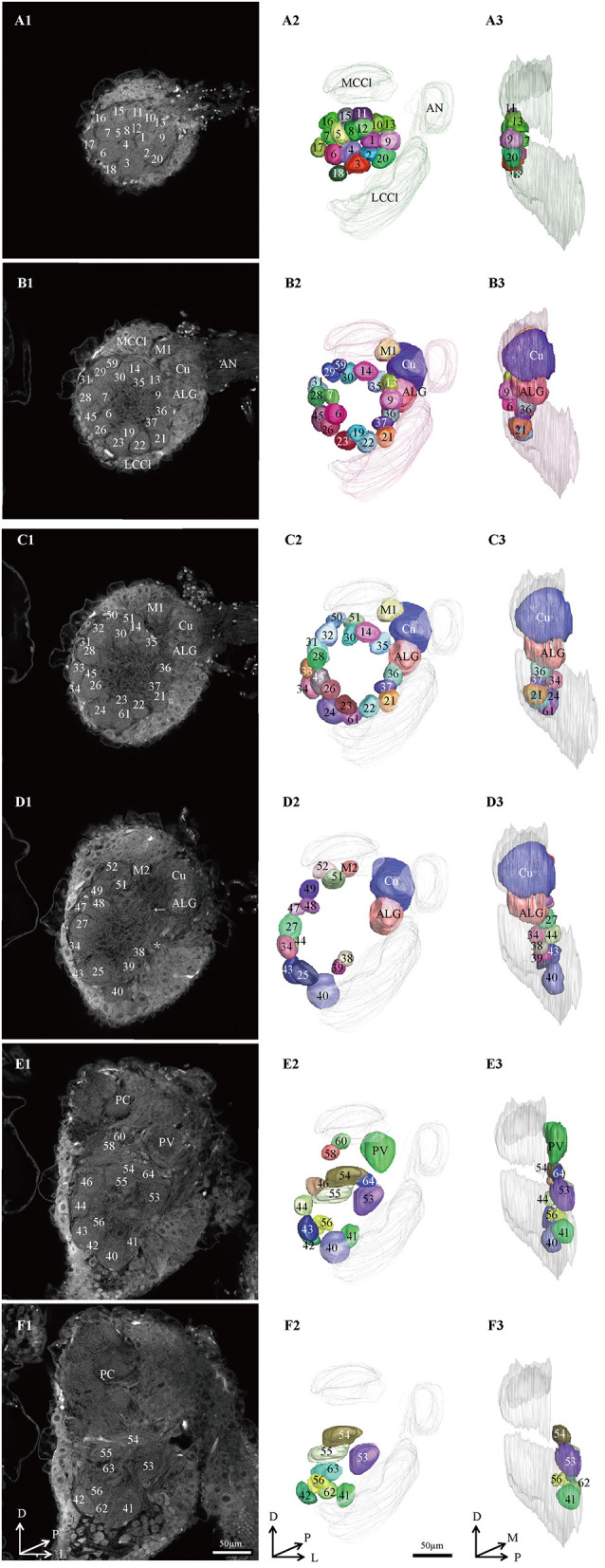
Confocal images and 3D reconstructions of the AL glomeruli of male *E. Grisescens* in a frontal view. **(A1–F1)** confocal images of AL sections at different depths. The most anterior glomeruli, at 13 μm (A), 33 μm (B), 46 μm (C), 72 μm (D), 90 μm (E), and 104 μm (F). **(A2–F2)** 3D reconstructions of glomeruli from the confocal images shown in **(A1–F1)** (frontal view). **(A3–F3)** lateral view of the 3D reconstructions shown in **(A2–F2)**. AN, antennal nerve; LCCl, lateral cell cluster; MCCl, medial cell cluster; PC, protocerebrum; Cu, cumulus; ALG, anterior-lateral glomerulus; M1, medial glomerulus 1; M2, medial glomerulus 2; PV, posterior-ventral glomerulus; A, anterior; D, dorsal; M, medial. Scale bars 50 μm. Images were drawn by AMIRA 5.3 and further enhanced by adobe illustrator CS 5.

Thirteen AL spheres from the brains of nine male *E. obliqua* and ten AL spheres from the brains of *eight* male *E. grisescens* were reconstructed in this study. In both species, the AL was positioned at the posterior deutocerebrum of the brain, as previously described for other species (Berg et al., 2002; Zhao et al., 2016). The exterior of the AL was similar in both species, except for the larger hemi-lateral sphere. The thickness of the AL was 143.371 ± 25.16 μm and 170.96 ± 38.10 μm in *E. obliqua* and *E. grisescens*, respectively (Table 1). One sphere of the AL was composed of many neuropil units of spheroidal shape, called glomerulus. All glomeruli were distributed in a demarcated layer surrounding the hub (Figures 1, 2). The lateral cell cluster (LCCl) and medial cell cluster (MCCl) were stained by both the antibody and PI (Figures 1, 2, **6I,K,L,N**). The LCCl and MCCl were positioned dorsal-medially and lateral-ventrally of the AL, respectively (Figures 1, 2). The average thickness of LCCl was larger than that of MCCl in both *E. obliqua* and *E. grisescens*. Although specimens of MCCl and LCCl were slightly thicker in *E. grisescens*, the ratio to AL thickness displayed no significant difference between the species (Table 1).

**Table 1:** Specimen thickness of lateral cell cluster (LCCL), medial cell cluster (MCCl), and antennal lobe (AL) in male *Ectropis. grisescens* and *Ectropis obliqua*.

**Specimen**		**MCCL thickness**	**LCCL thickness**	**AL thickness**	**MCCL**	**LCCL**	***n***
		**(μm/slices)**	**(μm/slices)**	**(μm/slices)**	**/AL**	**/AL**	
Male	Average	49.54/70.29	79.98/116	143.51/198.57	0.35	0.57	7
*E. obliqua*	SD	10.42/18.39	10.02/15	25.44/26.32	0.08	0.12	
Male	Average	62.91/94.83	101.95/154	170.96/257.67	0.39	0.60	6
*E. grisescens*	SD	10.88/16.42	24.45/37	38.10/57.19	0.12	0.10	

*Data were extracted by ZEN software. n indicated the number of specimens*.

### Identification of Glomeruli of the Whole Antennal Lobe in Male *E. obliqua* and *E. grisescens* and the Dimorphism Between Male and Female *E. grisescens*

**Figure 3:**
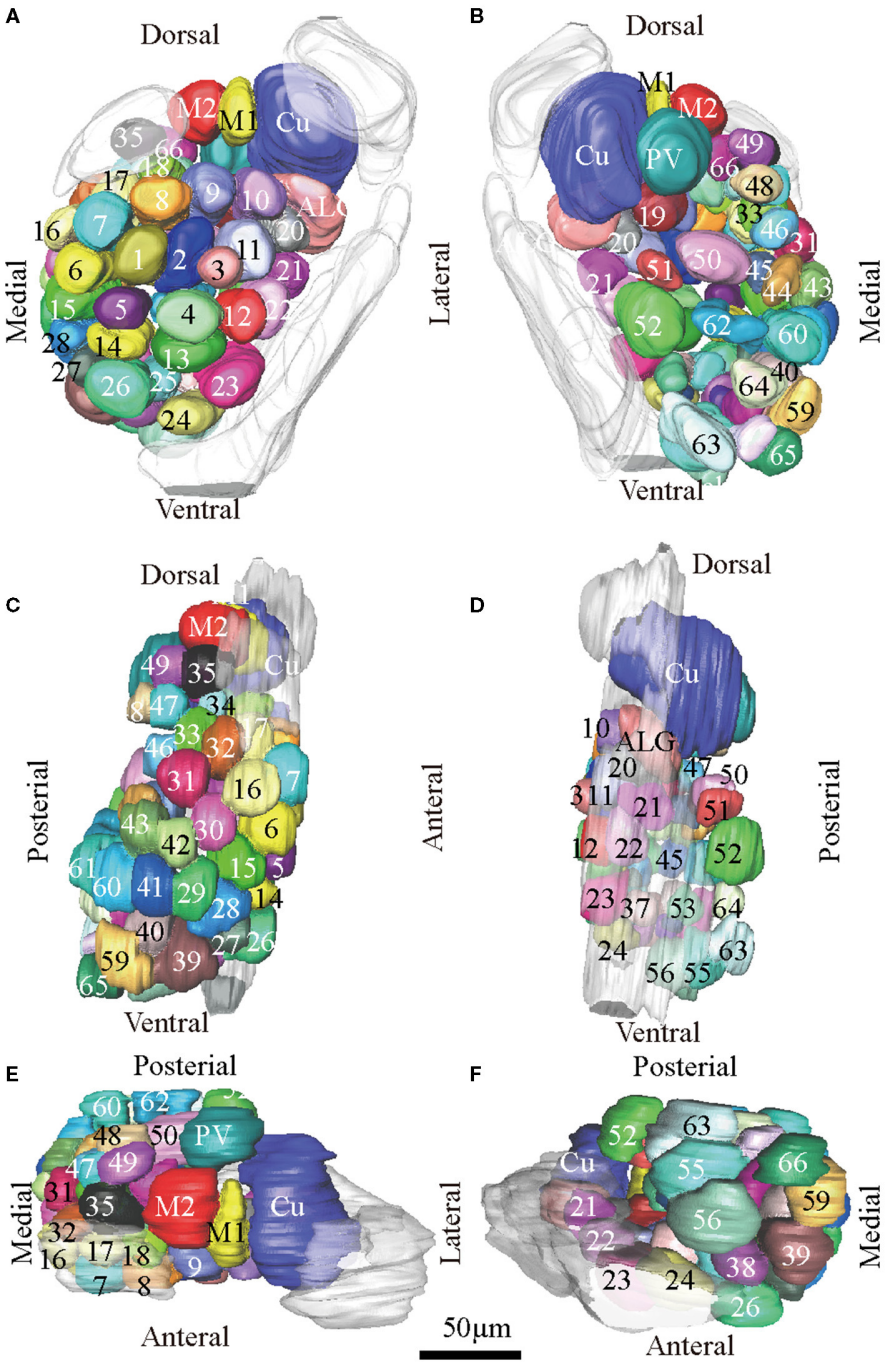
Atlas of the 3D reconstruction of the left AL of male *E. obliqua*. All glomeruli are reconstructed and given a specific number and color. **(A)** anterior view. **(B)** posterior view. **(C)** medial view. **(D)** lateral view. **(E)** dorsal view. **(F)** ventral view. Cu, cumulus; ALG, anterior-lateral glomerulus; M1, medial glomerulus 1; M2, medial glomerulus 2; PV, posterior-ventral glomerulus. Dotted outline indicated MGC area in **(A,B)**. Scale bar is 50 μm (applies to **A–F**). Images were drawn by AMIRA 5.3 and further enhanced by adobe illustrator CS 5.

**Figure 4:**
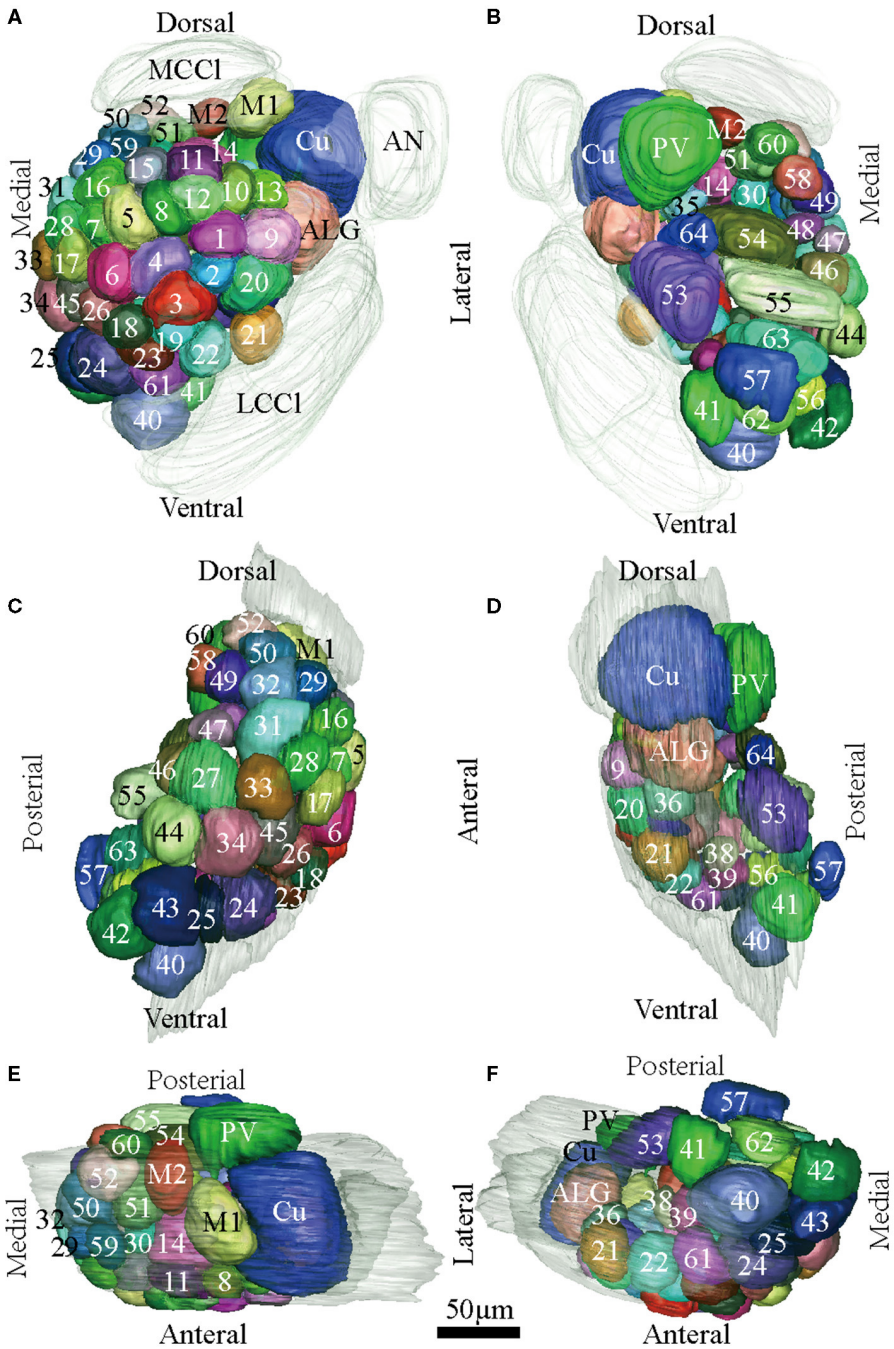
Atlas of the 3D reconstruction of the left AL of male *E. Grisescens*. All glomeruli are reconstructed and given a specific number and color. **(A)** anterior view. **(B)** posterior view. **(C)** medial view. **(D)** lateral view. **(E)** dorsal view. **(F)** ventral view. Cu, cumulus; ALG, anterior-lateral glomerulus; M1, medial glomerulus 1; M2, medial glomerulus 2; PV, posterior-ventral glomerulus. Dotted outline indicated MGC area in **(A,B)**. Scale bar is 50 μm (applies to **A–F**). Images were drawn by adobe illustrator CS 5. Images were drawn by AMIRA 5.3 and further enhanced by adobe illustrator CS 5.

**Figure 5:**
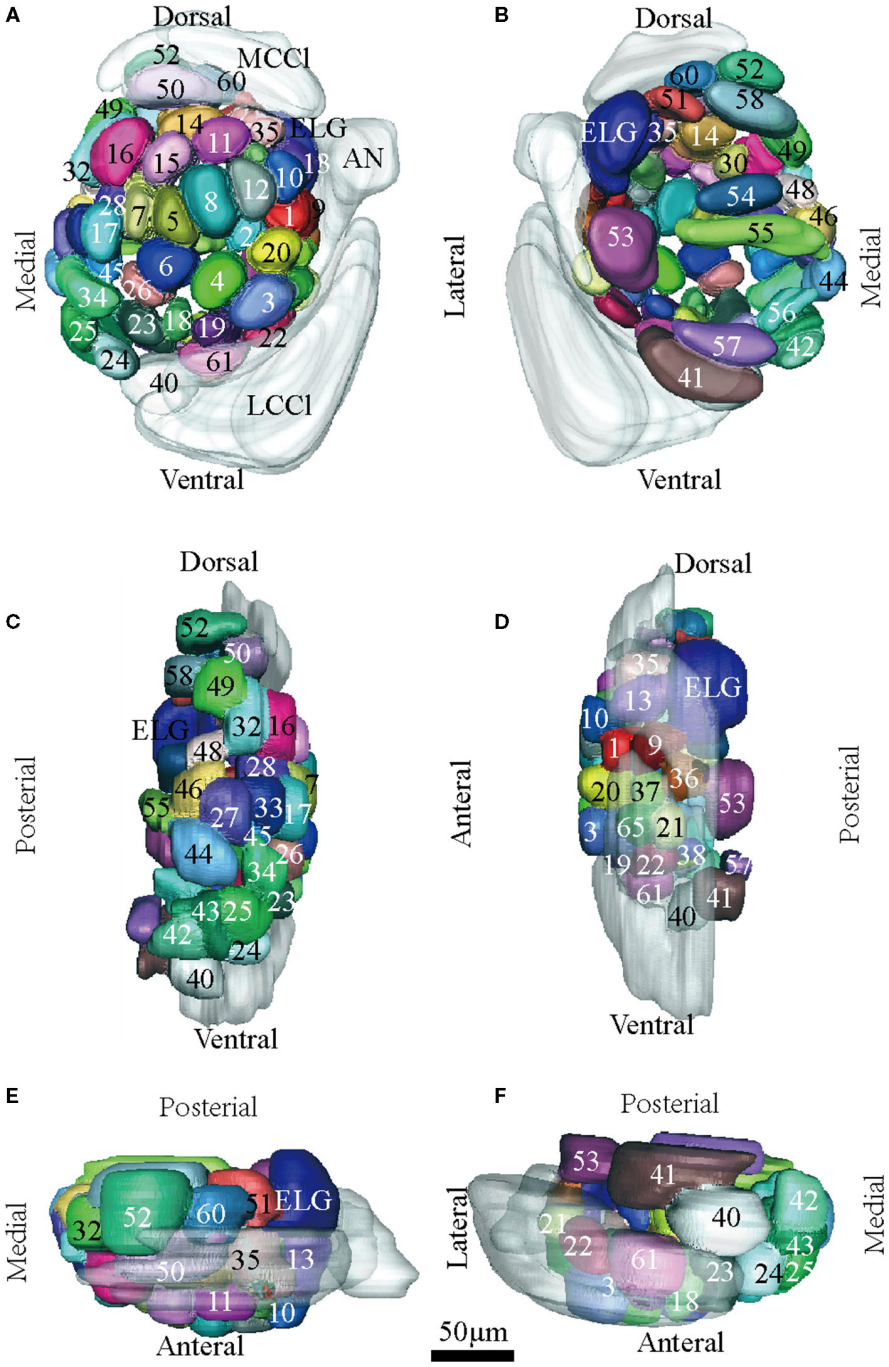
Atlas of the 3D reconstruction of the left AL of female *E. grisescens*. All glomeruli are reconstructed and given a specific number and color. **(A)** anterior view. **(B)** posterior view. **(C)** medial view. **(D)** lateral view. **(E)** dorsal view. **(F)** ventral view. ELG: enlarged glomerulus. Scale bar is 50 μm (applies to **A–F**). Images were drawn by adobe illustrator CS 5. Images were drawn by AMIRA 5.3 and further enhanced by adobe illustrator CS 5.

**Figure 6:**
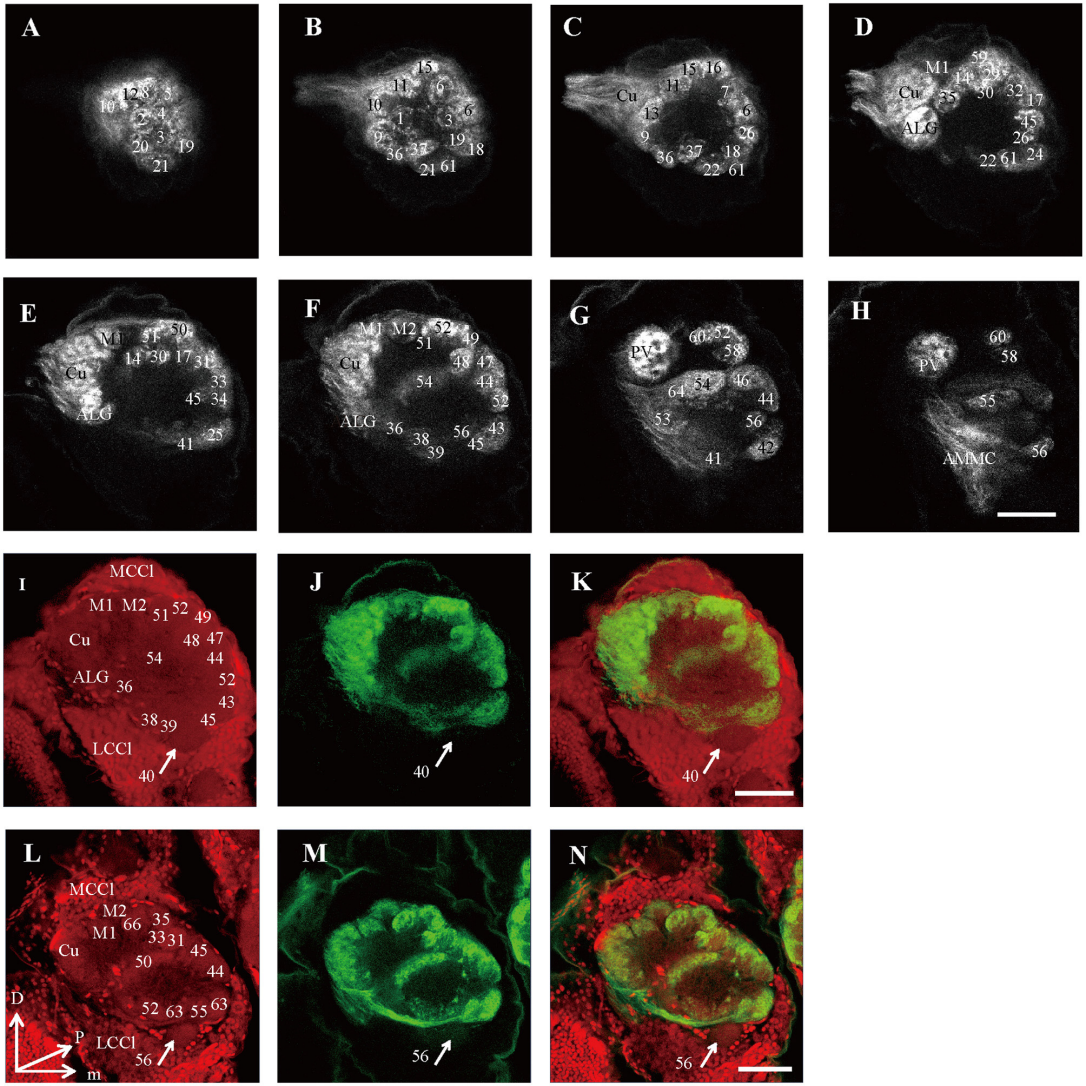
Confocal images showing OSNs projecting into the right AL of male *E. grisescens* and *E. obliqua*. Labeling was obtained by applying antennae backfill with PI co-staining. **(A–H)** confocal sections at different depths of AL showing OSNs innervating in male *E. Grisescens*. The most anterior glomeruli, at 26 μm (A), 37 μm (B), 46 μm (C), 59 μm (D), 72 μm (E), 85 μm (F), 105 μm (G), and 117 μm (H). **(I–K)** showed confocal images 85 μm with co-staining of antennae backfill with PI in male *E. grisescens*. Arrows indicated G40 receives no axons from OSNs. **(L–N)** showed confocal images 85 μm with co-staining of antennae backfill with PI in male *E. obliqua*; Arrows indicated G56 receives no axons from OSNs. LCCl, lateral cell cluster; MCCl, medial cell cluster; Cu, Cumulus; ALG, anterior-lateral glomerulus; M1, medial glomerulus 1; M2, medial glomerulus 2; PV, posterior-ventral glomerulus; D, dorsal; L, lateral; M, medial; V, ventral. Scale bars 50 μm in **(H)** applies to **(A–H)**; in **(K)** applies to **(I–K)**; in **(N)** applies to **(L–N)**. The images were drawn by AMIRA 5.3 and further enhanced by adobe illustrator CS 5.

In order to identify the male-specific glomeruli of *E. obliqua* and *E. grisescens*, we reconstructed the whole AL sphere of male *E. obliqua* and *E. grisescens* and female *E. grisescens*. The glomerulus of the AL was marked by Arabic numerals in a clockwise direction from anterior to posterior (Figures 1, 2). Four areas were easily recognized by the position and shape of the glomeruli and are described below. Area 1 was the entrance of the antennal nerve and contained a large glomerulus, called cumulus in male *E. obliqua* and *E. grisescens*. Around the cumulus were four smaller satellite glomeruli, and all five glomeruli were larger than the adjacent glomeruli in male *E. gresescens* and *E. obliqua*, therefore, they were primarily identified as the MGC and landmarked by the antennal nerve and cumulus (Figures 3, 4). Area 2 was the dorsal-medial area landmarked as G35 for *E. obliqua* (Figure 3) and G52 for *E. grisescens* (Figures 4, 5). This area was located at the dorsal edge of the AL and adjacent to M2. Area 3 was the ventral area, landmarked as G56, G59 for *E. obliqua* (Figure 3), and G40 and G42 for *E. grisescens* (Figures 4, 5). G59 and G42 were deeper stained by presynaptic antibody and PI, shown at the slices (Figures 1G1, 2F1), and G56 and G40 did not project from the ORNs dendrite (Figures 6J,M). Area 4 was the posterior area, landmarked as G50, G51, and G52 for *E. obliqua* (Figure 3) and G53, G54, and G64 for *E. grisescens* (Figures 4, 5). This area was situated at the posterior edge of the AL, and G50 and G53 were clavate (Landmarks were shown in Table 2). Glomeruli of these four areas were identified according to the relative position of the landmarks and the others were recognized according to their relative position to the identified glomeruli.

**Table 2:** Primary landmarks for identification of Ordinary Glomeruli (OG) in male *E. obliqua* and *E. grisescens* and female *E. grisescens*.

	**Landmarks**	**Specific characteristics of the landmarks**	**Identified glomeruli according to the relative position of the landmarks**
Male and female	Antennal nerve	Largest bundle of the specimen	Cumulus, M1, M2, PV, ALG/ELG
*E. grisescens*	G52	At the dorsal edge of AL and adjacent to M2	G47, G48, G49, G50, G51, G58, G60
	G54	Clavate at the posterior edge of AL	G46, G27, G44, G34, G33, G31, G32, G28, G45, G17, G6, G26, G23, G18
	G53, G64	At the posterior edge and adjacent to ALG and G54	G9, G20, G21, G22, G36, G37, G38, G39, G61
	G40	LPOG, at anterior-lateral of AL and adjacent to G42	G41, G57, G38, G39
	G42	At posterior-ventral of AL and deeply stained	G43, G25, G24
Male	Antennal nerve	Largest bundle of the specimen	Cumulus, M1, M2, PV, ALG
*E. obliqua*	G35	At the dorsal edge of AL and adjacent to M2	G31, G46, G47, G34, G66, G48, G49
	G51	Clavate at the posterior edge of AL	G44, G45, G43, G60, G41, G42, G31, G32, G30, G29, G15, G14, G28, G26, G27
	G50, G52	At the posterior edge and adjacent to ALG and G51	G20, G11, G23, G24, G21, G22, G45, G53, G25
	G56	LPOG, at anterior-lateral of AL and adjacent to G65	G55, G63, G45, G53
	G65	At posterior-ventral of AL and deeply stained	G59, G40, G39

*Cu, cumulus; ALG, anterior-lateral glomerulus; M1, medial glomerulus 1; M2, medial glomerulus 2; PV, posterior-ventral glomerulus. The landmarks were chosen based on the most obvious glomeruli in terms of position and shape*.

There was clear dimorphism between female and male *E. grisescens*. The glomeruli number and volume of the female *E. grisescens* AL was smaller than that of males (Tables 3, 4). Only one enlarged glomerulus (ELG) existed at the entrance of the antennal nerve in female *E. grisescens* whose volume was much smaller than the cumulus. The shape of G50 and G58 at the dorsal-medial area of the female AL was clavate (Figure 5). G17, G21, G22, G24, G25, G27, G30, G32, G38, G39, G40, G42, G43, and G45 were larger in males; however, G50 and G58 were larger in females (Table 4). G 29, G31, G48, G59, G62, G63, and G64 were not detected in female *E. grisescens*, and the two other glomeruli, G65 and G66, were absent in male *E. grisescens*. Most glomeruli in the ventral-medial area of the AL and at the entrance of the antennal nerve of females were smaller than those in males. Therefore, the appearance of the AL sphere in females was almost spherical and that of males was fusiform, in the frontal view (Figures 4, 5).

**Table 3:** Comparison of number and volume of the glomerulus in male *E. obliqua*, male *E. grisescens*, and female *E. grisescens*.

**Specimen**	**Total number**	**Number of glomeruli possessing distinct volumes**				**Total volume (10^**3**^ μm)**	**Average volume (10^**3**^ μm)**	***n***
		** <5 (10^**3**^ μm)**	**5–10 (10^**3**^ μm)**	**10–30 (10^**3**^ μm)**	**40 (10^**3**^ μm)**			
Male*E. obliqua*	67	12	36	19	0	552.7	8.25	1
Male*E. grisescens*	64	0	26	35	3	801.72	12.53	4
Female*E. grisescens*	59	5	32	22	0	617.00	10.28	4

*n indicated the number of specimens*.

**Table 4:** Comparison of the OG volume between male and female *E. grisescens*.

	**MEG**			**FEG**		
	**Average**	**SD**	***n***	**Average**	**SD**	***N***
G1	9,319.68	1,990.84	4	10,019.13	3,065.37	4
G2	9,755.57	3,452.96	4	4,873.79	1,359.08	3
G3	12,034.59	4,197.75	4	10,053.35	1,572.98	4
G4	9,314.29	813.44	4	9,592.41	1,756.35	4
G5	10,898.70	4,738.37	4	7,276.96	2,276.79	4
G6	14,293.66	3,961.40	4	10,209.65	626.96	4
G7	8,583.62	609.74	3	7,912.36	2,121.56	4
G8	9,603.40	4,170.71	4	9,698.57	762.03	4
G9	12,738.10	2,768.74	4	9,388.99	4,958.54	4
G10	9,249.31	1,914.87	4	9,039.34	5,637.08	4
G11	10,752.71	1,533.83	4	9,903.65	4,022.17	4
G12	6,720.91	1,715.90	4	7,104.07	1,886.99	4
G13	7,855.77	1,204.40	4	9,810.85	2,770.41	3
G14	13,868.06	4,202.53	4	9,177.46	1,401.56	4
G15	6,875.46	1,143.32	4	9,553.45	2,130.97	4
G16	11,268.65	4,175.97	4	10,830.82	2,926.24	4
G17	10,773.38	4,104.55	4	6,796.03	1,921.72	4
G18	8,475.12	4,840.34	4	8,496.06	2,750.01	4
G19	9,815.03	788.27	4	11,240.36	5,401.28	4
G20	7,125.62	5,742.90	4	10,802.29	2,210.49	4
G21	14,889.89	619.12	4	5,725.85	1,564.62	4
G22	14,976.01	176.27	4	7,084.03	3,445.05	4
G23	9,700.39	2,040.60	4	6,148.79	1,808.32	4
G24	21,438.90	6,290.17	4	6,935.56	1,329.04	4
G25	19,940.78	6,175.03	4	7,586.45	4,110.94	4
G26	11,577.47	3,829.61	4	11,427.15	2,907.94	4
G27	15,152.34	2,686.88	4	9,940.22	5,312.82	4
G28	10,626.33	1,909.44	4	10,095.43	2,693.00	4
G29	7,403.25	1,734.03	2			
G30	9,415.86	2,101.34	3	3,576.23	356.34	2
G31	12,740.29	3,707.55	4			
G32	14,063.10	2,889.18	4	6,168.54	3,441.69	4
G33	10,935.79	3,943.30	4	6,697.45	2,042.14	4
G34	14,024.20	3,148.78	4	9,205.87	944.05	4
G35	9,877.63	328.62	3	8,717.74	2,385.97	4
G36	9,621.80	937.46	4	7,141.27	1,973.17	4
G37	9,080.68	2,410.58	4	5,610.91	3,198.97	4
G38	7,621.70	2,305.69	4	1,957.04	296.32	3
G39	6,751.85	1,662.03	4	2,050.47	865.39	3
G40	33,311.61	4,124.21	4	21,897.09	3,425.65	4
G41	26,594.96	10,767.63	4	25,380.78	3,933.29	4
G42	20,089.42	6,671.27	4	11,174.09	3,517.32	4
G43	24,377.49	7,886.46	4	10,289.70	2,476.03	3
G44	15,524.32	4,766.72	4	10,056.64	1,733.10	4
G45	12,354.68	4,104.04	4	6,083.66	1,962.81	4
G46	10,772.03	4,132.44	4	10,925.79	3,756.51	4
G47	5,832.04	2,502.49	4	4,724.82	1,494.10	4
G48	8,604.37	1,455.06	3			
G49	11,275.30	2,177.53	4	8,236.54	717.53	4
G50	10,675.90	3,140.79	4	13,042.97	1,355.20	4
G51	9,096.62	3,555.25	4	7,094.66	4,112.04	4
G52	11,064.42	1,341.16	4	12,244.61	1,602.26	4
G53	35,535.36	5,474.28	4	29,785.49	9,158.49	4
G54	33,836.40	4,296.80	4	26,240.16	11,483.42	4
G55	28,069.16	4,949.88	4	19,151.33	6,653.85	4
G56	10,993.84	5,471.15	4	7,235.92	1,842.36	4
G57	16,681.20	4,720.03	4	13,483.03	5,753.61	4
G58	8,191.81	2,351.63	4	16,002.94	2,981.00	4
G59	6,435.56	2,212.66	3			
G60	12,550.87	4,236.84	4	9,391.89	3,660.24	4
G61	12,530.05	4,177.33	4	12,225.45	3,424.02	4
G62	14,223.78	2,941.83	3			
G63	18,595.43	5,033.31	4			
G64	8,777.96	5,230.68	2			
G65				5,067.86	1,152.36	4
G66				5,556.99	1,935.64	4
ELG				33,857.05	6,950.06	4

*The enlarged glomerulus (ELG) indicated the enlarged glomerulus in female E. grisescens and n indicated the number of specimens*.

### Innervation of Olfactory Receptor Neurons Into the Antennal Lobe in *E. obliqua* and *E. grisescens*

Because presynaptic antibody staining applied to *E. grisescens* was not as successful as that applied to *E. obliqua* (Figure 2), we applied antennae backfill as an alternative method to confirm the results of *E. grisescens*. Antenna backfill with PI co-staining was used to display the innervating of ORNs. These results showed that OSNs of both *E. obliqua* and *E. grisescens* innervated almost the whole AL, except for G56 of *E. obliqua* and G40 of *E. grisescens* (Figure 6).

### Comparison of the Macroglomerular Complex Between *E. obliqua* and *E. grisescens*

**Figure 7:**
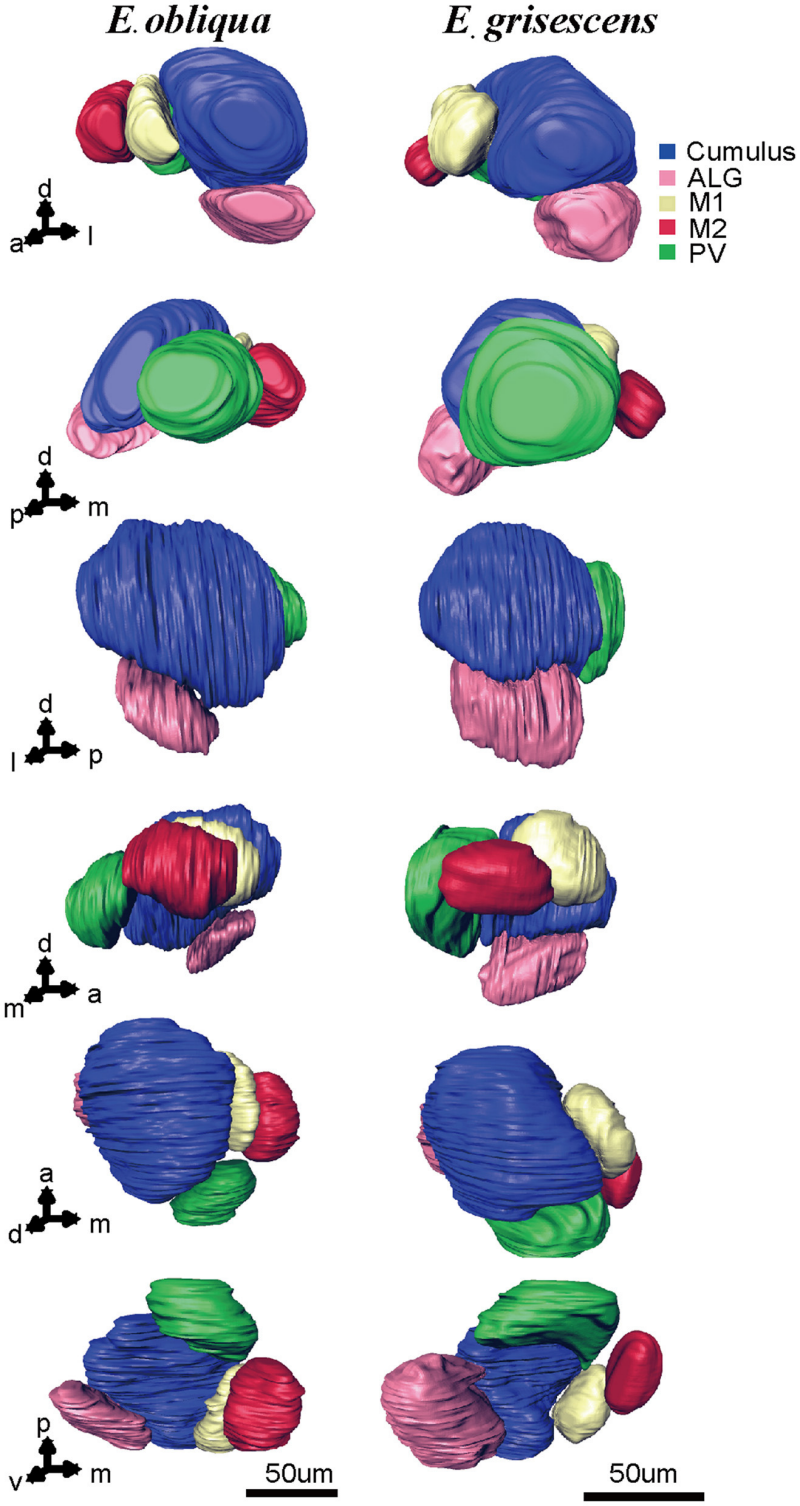
Atlas of the 3D reconstruction of the MGC of male *E. grisescens* and *E. obliqua* exacted from Figures 3, 4. Arrows 1–6 show the anterior view, posterior view, lateral view, medial view, dorsal view, and ventral view, respectively. The colored portions are as follows: Cumulus, blue; ALG, pink; M1, yellow; M2, red; Posterior-ventral glomerulus (PV), green. The images were drawn by AMIRA 5.3 and further enhanced by adobe illustrator CS 5.

According to the comparison of glomeruli of female *E. grisescens*, male-specific MGC of *E. grisescens* was identified at the entrance of the AL and was, evidently larger than the other glomeruli (Figures 1–4). Because no difference was detected in the general morphology of glomeruli between *E. obliqua* and *E. grisescens* in this study, the MGC of *E. obliqua* was defined in the same way as that of *E. grisescens*. In both species, the MGC was composed of five glomeruli, similar to that in other species: cumulus (Cu), anterior-lateral glomerulus (ALG), medial glomerulus 1 (M1), medial glomerulus 2 (M2), and posterior-ventral glomerulus (PV) (Figures 1–4, 7). The position of each glomerulus was the same in both species (Figures 1–4, 7). The largest glomerulus, Cu, was at the entrance of the antennal nerve and chosen as a landmark of the MGC (Figures 1–4).

### Volume Difference of Macroglomerular Complex Glomeruli Between *E. obliqua* and *E. grisescens*

**Figure 8:**
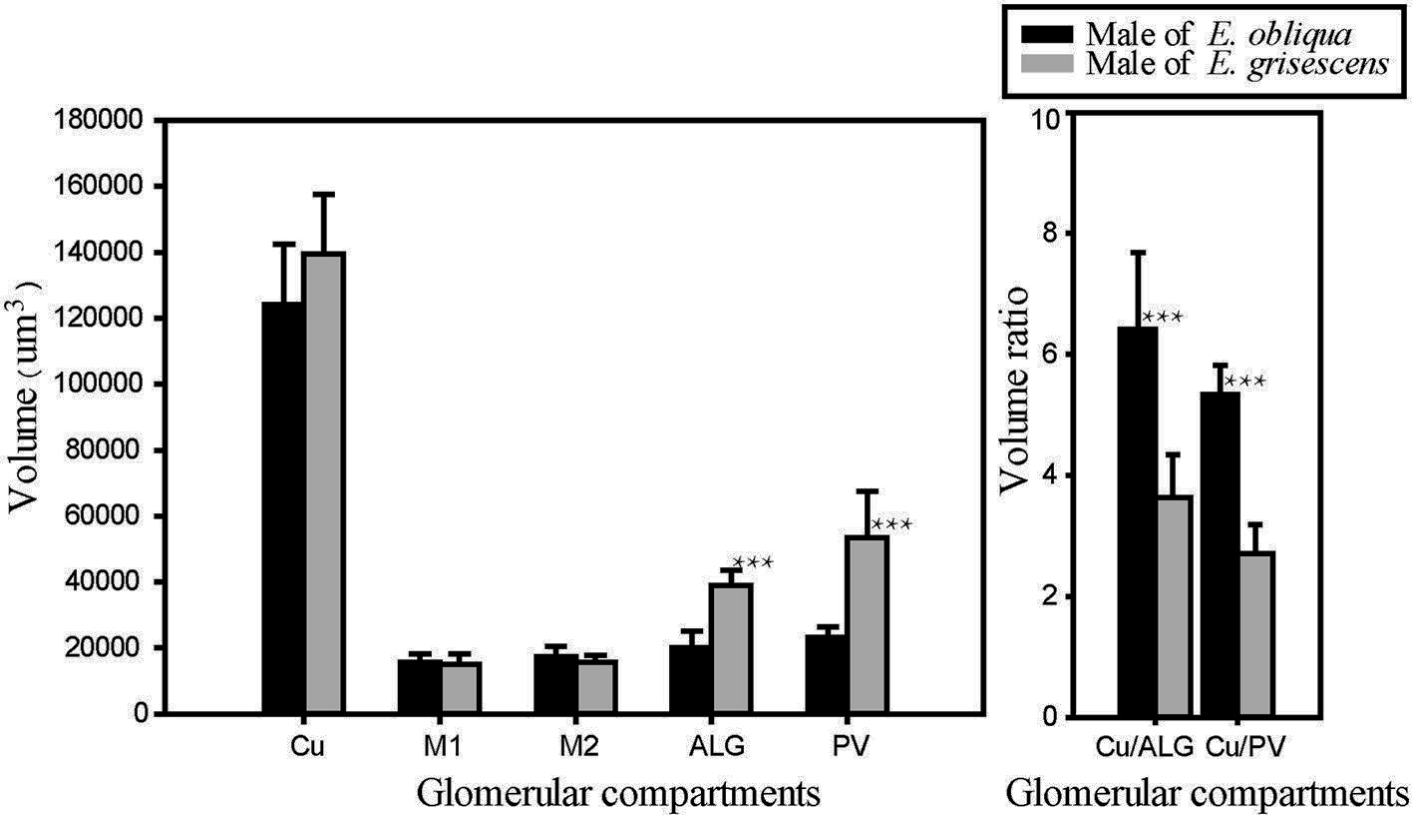
Comparison of MGC volume between male *E. obliqua* and *E. grisescens*. The data is analyzed by one-away ANOVA and *t*-test in SigmaPlot. The plotted values are mean ± SD. ^***^P ≤ 0.001., *n* is 10 and 13, respectively, in *E. obliqua* and *E. grisescens*. The images were drawn by SigmaPlot Portable and further enhanced by adobe illustrator CS 5.

**Figure 9:**
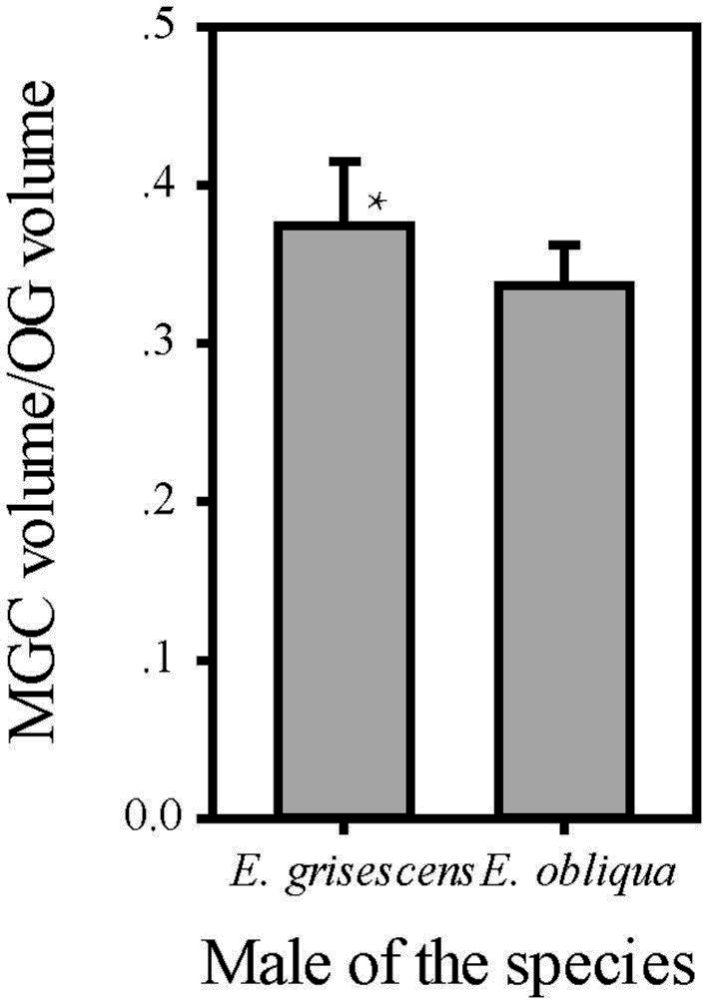
Comparison of ratio of MGC volume to OG between male *E. obliqua* and *E. grisescens*. The data is analyzed by one-away ANOVA. The plotted values are mean ± SD. *P* = 0.049. *indicates significant difference. “*n*” is 7 and 9, respectively, in *E. obliqua* and *E. grisescens*. The images were drawn by SigmaPlot Portable and further enhanced by adobe illustrator CS 5.

The volume of each glomerulus within the MGC was calculated for *E. obliqua* and *E. grisescens*. The results revealed that ALG and PV were larger in *E. grisescens* than in *E. obliqua* [ALG: 37,884 ± 3,529 μm^3^ (*n* = 10) and 19,010 ± 4,445 μm^3^ (*n* = 13), respectively; PV: 42,088 ± 8,815 μm^3^ (*n* = 10) and 22,098 ± 2,615 μm^3^ (*n* = 13), respectively]. The volumes of other glomeruli were not statistically different between the two species (Table 5, Figure 8). The volume ratios of Cu to ALG and PV were higher in *E. grisescens* than those in *E. obliqua* (Figure 8). The MGC proportion, which was calculated as the total volume ratio of MGC to ordinary glomeruli (OG), was smaller in *E. obliqua* than that in *E. grisescens* (0.33 ± 0.03 and 0.37 ± 0.04, respectively; Figure 9).

**Table 5:** Comparison of volume of the MGC glomerulus in male *E. obliqua* and *E. grisescens*.

	**Male** ***E. obliqua***			**Male** ***E. grisescens***		
	**Volume (μm^**3**^)**	**SD**	***n***	**Volume (μm^**3**^)**	**SD**	***n***
Cu	116,899	16,369	10	123,463	7,477	13
ALG	19,010	4,445	10	37,884	3,529	13
M1	16,556	1,390	10	12,956	651	13
M2	17,464	2,882	10	14,259	1,350	13
PV	22,098	2,615	10	42,088	8,815	13

*n indicated the number of specimens*.

## Discussion

The MGC is regarded as the olfactory center of insects for sex pheromone sensing, and it is present in male moths (Galizia et al., 2000; Hansson and Anton, 2000; Zhao et al., 2016). Studies on the coding mechanism of sex pheromone blends in the MGC between sibling species have focused on noctuid moths. Generally, closely related species contain the same number of glomeruli in the MGC. The Cu is the largest glomerulus, and it receives innervation from ORNs tuned to the main pheromone component of each species. The other glomeruli are smaller than Cu and receive information on minor pheromone components or inhibition chemicals (Berg et al., 2014). Differences in the positions of a single glomerulus have been shown between two *Helicoverpa* species, *H. armigera* and *H. assulta* (Xu et al., 2016a,b; Zhao et al., 2016), but not between *Heliothis virescens* and *H. subflexa* (Vickers and Christensen, 2003). In this study, the same number and position of glomeruli were found in the MGC between *E. obliqua* and *E. grisescens* (Figure 7), similar to those of *Heliothis* species. However, the volumes of ALG and PV were larger in *E. grisescens* than in *E. obliqua* (Figure 8; Table 5). The antennae sensilla of both ORNs are reflected in the volume of the corresponding glomerulus, so that species can detect several intra- and interspecies sex pheromone components (Liu et al., 2019). However, the population density of ORNs for sensing the minor component in the pheromone blends was higher in *E. grisescens* than in *E. obliqua*. Therefore, our results support the premise that the number of ORNs is reflected in the volume of the corresponding glomeruli (Christensen and Hildebrand, 1987; Hansson and Anton, 2000; Masse et al., 2009; Kuebler et al., 2012; Brill et al., 2013), which has proven to be important in peripheral coding in closely related *Helicoverpa* species (Wu et al., 2013, 2015). Therefore, it is reasonable to propose that ALG or PV might be responsible for detecting the minor component Z3, Z6,Z9-18:H in both *E. obliqua* and *E. grisescens*. One of the medial glomeruli (M1 or M2) might be related to the specific component Z3,epo6,Z9-19:H of *E. obliqua*, but functional evidence is needed.

The difference in the ratio of minor to main sex pheromone components might be an important olfactory coding mechanism in closely related species. Noctuid species that are sympatric and synchronic generally contain different main or minor components or different combinations of the same components (Berg et al., 2014). Therefore, differences in the coding for the minor component might not be apparent. In the sibling species *H. armigera* and *Helicoverpa. zea*, both use Z11-16:AL as the main component and Z9-16:AL as the minor component with a ratio of 100:5(20:1) and 98:2, respectively, (Kehat and Dunkelblum, 1990; Fadamiro and Baker, 1997). In both species, the ORNs tuned to Z9-16:AL and received innervation from the ALG glomerulus of the respective MGC (Lee et al., 2006; Xu et al., 2016a,b), similar to the pattern that we observed in *E. obliqua* and *E. grisescens*. Thus, this coding mechanism in closely related species might be a common feature among moth species. No earlier study on these two species has focused on this coding mechanism because *H. armigera* and *H. zea* are not regarded as sympatric species (Cunningham and Zalucki, 2014; Mastrangelo et al., 2014; Anderson et al., 2016).

According to available information on the ALs of moth species, those in the same group contain specific and consistent patterns of MGC arrangement. For example, noctuid moths contain three to four MGC compartments, of which the Cu is the largest (Skiri et al., 2005; Berg et al., 2014; Xu et al., 2016a,b). Bombycid moths display different volume combinations of cumulus to toroid (Namiki et al., 2014). In this study, five glomeruli were found in both species with invariant position and number. These results also showed that the arrangement of MGC compartments in these species is the same as that in noctuid species, with Cu being the largest glomerulus, which might be consistent among Geometridae species.

This study also showed that MGC to OG proportion was 0.33 ± 0.03 and 0.37 ± 0.04 in *E. obliqua* and *E. grisescens*, respectively (Figure 9). The slightly larger MGC in *E. grisescens* than in *E. obliqua* might result from the larger ALG and PV in *E. grisescens*. The MGC proportion was close to that of the diamondback moth, *Plutella xylostella* (0.32), and much higher in both *E. obliqua* and *E. grisescens* than in *H. armigera* (0.12) (Skiri et al., 2005; Zhao et al., 2016; Yan et al., 2019). The significant difference in MGC size might reflect the higher level of specialization in the sex pheromone olfactory processing of both *E. obliqua* and *E. grisescens*.

Except the few species containing only one MGC component (Varela et al., 2009), moth species producing Type I pheromones generally contain three to four glomeruli in their MGC (Hansson et al., 1994; Lee et al., 2006; Namiki et al., 2014; Nirazawa et al., 2017; Yan et al., 2019). It is notable that in both moth species studied here, producing Type II pheromones, five glomeruli were present in their MGC. Another pheromone Type II species, *Eilema japonica*, contains four glomeruli in its MGC (Namiki et al., 2012). The number of glomeruli in the MGC for the known moth species is summarized in Table 6. Overall, more glomeruli appear to be present in the MGC of pheromone Type II than in Type I species, suggesting that pheromone Type II species process more complex olfactory information during sex pheromone reception than pheromone Type I species. Given that ORNs, especially the ones tuned to inhibitory chemicals, display broadly innervating destinations in MGC glomeruli (Berg et al., 2014), the pheromone Type II species might be more evolved than pheromone Type I species regarding pheromone reception. However, further studies are needed to confirm this hypothesis.

**Table 6:** The MGC glomeruli number in different moth species.

**Sex pheromone types**	**Species**	**Number of MCGs**	**References**
Type I	*Heliothis virescens*	4	Vickers and Christensen, 2003
	*Heliothis subflexa*	4	Vickers and Christensen, 2003
	*Helicoverpa zea*	3	Lee et al., 2006
	*Helicoverpa assulta*	3	Berg et al., 2002
	*Helicoverpa armigera*	3	Zhao et al., 2016
	*Cydia molesta*	1	Varela et al., 2009
	*Plutella xylostella*	3	Yan et al., 2019
	*Mythimna separata*	3	Jiang et al., 2019
	*Athetis dissimilis*	3	Dong et al., 2020
	*Agrius convolvuli*	3	Nirazawa et al., 2017
	*Ernolatia moorei*	2	Namiki et al., 2014
	*Trilocha varians*	2	Namiki et al., 2014
	*Manduca sexta*	3	Rospars and Hildebrand, 2000
	*Rondotia menciana*	4	Namiki et al., 2014
	*Bombyx mandarina*	4	Namiki et al., 2014
	*Bombyx mori*	4	Namiki et al., 2014
Type II	*Eilema japonica*	4	Namiki et al., 2012
	*Ectropis obliqua*	5	
	*Ectropis grisescens*	5	

The presynaptic antibody staining for marking the AL glomeruli of *E. obliqua* and *E. grisescens* was not a suitable technique. The boundaries of certain single glomeruli were not clear when compared to those of other species when the same technique was used. If the contact time between the specimen and the antibody is prolonged, the glomeruli become clearer but the ORNs combined staining (antennae backfilled) failed to stain the glomeruli. The PI staining with the neurobiotin backfilled method seemed to overcome this difficulty (Figure 6). However, this method must be combined with antennae backfills, as PI was specifically developed to stain nuclei; by making the fiber lighter, the confocal images become less clear (Figure 6). Thus, a new method for specifically staining the AL glomeruli is still necessary for some species. Additionally, antennae backfill marking exhibited an innovative feature of the ORNs. The results showed that ORNs of both *E. obliqua* and *E. grisescens* innervated nearly the whole AL, except for G56 of *E. obliqua* and G40 of *E. grisescens* (Figure 5). Therefore, G56 of *E. obliqua* and G40 of *E. grisescens* were predicted as the labial pit organ glomerulus as found in previous studies, i.e., G38 in *H. armigera* (Zhao et al., 2016) and PV1 in *P. xylostella* (Yan et al., 2019).

## Data Availability Statement

The data used to support the findings of this study are available from the corresponding author upon request.

## Author Contributions

JL, Z-qL, and Z-mC conceived and designed the study and wrote the manuscript. Z-xL, X-mC, and LB provided the insects. JL, X-mC, LB, and KH analyzed and interpreted the data. All authors contributed to the article and approved the submitted version.

## Conflict of Interest

The authors declare that the research was conducted in the absence of any commercial or financial relationships that could be construed as a potential conflict of interest.

## Publisher's Note

All claims expressed in this article are solely those of the authors and do not necessarily represent those of their affiliated organizations, or those of the publisher, the editors and the reviewers. Any product that may be evaluated in this article, or claim that may be made by its manufacturer, is not guaranteed or endorsed by the publisher.
